# Development of a non-human primate BCG infection model for the evaluation of candidate tuberculosis vaccines

**DOI:** 10.1016/j.tube.2017.11.006

**Published:** 2018-01

**Authors:** Stephanie A. Harris, Andrew White, Lisa Stockdale, Rachel Tanner, Laura Sibley, Charlotte Sarfas, Joel Meyer, Jonathan Peter, Matthew K. O'Shea, Zita-Rose Manjaly Thomas, Ali Hamidi, Iman Satti, Mike J. Dennis, Helen McShane, Sally Sharpe

**Affiliations:** a Jenner Institute, Nuffield Department of Medicine, University of Oxford, Oxford, OX3 7DQ, UK; b Public Health England, Salisbury, SP4 0JG, UK

**Keywords:** Non-human primate, BCG infection, Tuberculosis, Vaccine

## Abstract

The lack of validated immunological correlates of protection makes tuberculosis vaccine development difficult and expensive. Using intradermal bacille Calmette-Guréin (BCG) as a surrogate for aerosol *Mycobacterium tuberculosis* (*M.tb*) in a controlled human infection model could facilitate vaccine development, but such a model requires preclinical validation. Non-human primates (NHPs) may provide the best model in which to do this. Cynomolgus and rhesus macaques were infected with BCG by intradermal injection. BCG was quantified from a skin biopsy of the infection site and from draining axillary lymph nodes, by culture on solid agar and quantitative polymerase chain reaction. BCG was detected up to 28 days post-infection, with higher amounts of BCG detected in lymph nodes after high dose compared to standard dose infection. Quantifying BCG from lymph nodes of cynomolgus macaques 14 days post-high dose infection showed a significant reduction in the amount of BCG detected in the BCG-vaccinated compared to BCG-naïve animals. Demonstrating a detectable vaccine effect in the lymph nodes of cynomolgus macaques, which is similar in magnitude to that seen in an aerosol *M.tb* infection model, provides support for proof-of-concept of an intradermal BCG infection model and evidence to support the further evaluation of a human BCG infection model.

## 1 Introduction

Tuberculosis (TB) remains an important global health threat, with an estimated 10.4 million new cases and 1.8 million deaths in 2015 [1]. A third of the world's population are latently infected with the causative agent, *Mycobacterium tuberculosis,* (*M.tb*) with a 10% lifetime risk of developing active disease, which increases in those co-infected with HIV [1]. This level of infection, combined with the emergence of multiple and extensively drug-resistant strains of *M.tb* [2], mean that an effective vaccine is an essential tool in controlling the spread of infection and reducing the burden of this disease.

Bacille Calmette-Guréin (BCG) is a live, attenuated strain of *Mycobacterium bovis* (*M. bovis*) and the only licenced TB vaccine. BCG confers variable levels of protection against pulmonary TB in adults, ranging from 0 to 80% depending on geographical location [3]. The lack of validated correlates of protection makes vaccine development very challenging, as it is difficult to predict whether a candidate vaccine will show efficacy in the target population without costly and time-consuming efficacy trials. A clinically advanced candidate vaccine, MVA85A, showed some promising results in pre-clinical animal models [4], [5], [6] and in early phase I/IIa safety and immunogenicity studies [7], [8], [9], but failed to show an improvement in efficacy over BCG alone in a large scale phase IIb trial in South African infants [10]. In light of this efficacy data, animal models used for TB vaccine development are being re-evaluated [11]. The use of controlled human infection models has greatly benefitted vaccine selection and development for other infectious diseases [12], [13], [14], [15]. Infecting humans with *M.tb* is not ethically acceptable, but the use of intradermal (ID) BCG as a surrogate for *M.tb* infection in a human mycobacterial infection model could aid in assessing potential efficacy at an early stage and in candidate vaccine selection. We have previously shown that a controlled human BCG infection model, where healthy adult volunteers receive BCG by intradermal administration into the upper arm and bacterial load is quantified from a biopsy of the site two weeks later, can detect a reduction in the amount of BCG recovered in previously BCG-vaccinated volunteers compared to those who were BCG-naïve [16], [17]. Further work has been conducted to optimise this infection model in humans, by varying strain and dose of BCG used [18].

This ID BCG infection model has evidence of biological validity as a surrogate for aerosol *M.tb* infection as both infection models have demonstrated a similar BCG vaccine effect in mice [19]. Minassian et al. showed that the efficacy of BCG vaccination against subsequent BCG infection of the skin in the murine model was comparable to the known efficacy of BCG against *M.tb* infection in the lung. In a similar BCG infection model in cattle, Villarreal et al. demonstrated that infecting previously BCG-vaccinated animals intranodally with BCG Tokyo showed partial protection compared to BCG-naïve animals, paralleling the level of BCG efficacy seen with aerosol *M. bovis* infection [20]. Non-human primates (NHPs) are considered to provide the most representative animal model for TB vaccine studies, due to their anatomical and physiological likeness to humans and similar pathology and infection outcome [21], [22]. BCG vaccination is known to be partially protective against aerosol *M.tb* infection in both cynomolgus and rhesus macaques [23], [24]. Both species have been used extensively in research into the pathogenesis of *M.tb* infection and the pre-clinical evaluation of candidate TB vaccines [22]. As it is not possible to compare an ID BCG infection model with an aerosol *M.tb* infection model in humans, demonstration of a similar BCG vaccine effect in the two models in NHPs would bridge this gap and provide biological validation for use of this model in humans for the evaluation of new candidate TB vaccines.

Here we present work carried out in NHPs to develop this BCG infection model in cynomolgus and rhesus macaques. We started with a study based on the design of successful early clinical trials with the BCG infection model in humans [16], [17] and then developed this model further for use in NHPs. If validated against aerosol *M.tb* infection, this BCG infection model would also provide a less expensive, less severe method of testing candidate TB vaccines in NHPs. It would not require the use of expensive BSL3 containment facilities and would greatly enhance the welfare of the animals [25], in line with the ‘refinement’ criteria of the National Centre for Replacement, Refinement and Reduction of Animals in Research (NC3Rs) [26], [27].

## 2 Methods

### 2.1 Experimental animals

The animals used in these studies were rhesus macaques of Indian genotype and cynomolgus macaques of Mauritian genotype obtained from established UK breeding colonies and were between 4 and 15 years of age. Absence of previous exposure to mycobacterial antigens was confirmed by a tuberculin skin test and screening using an *ex-vivo* IFN-γ ELISpot (MabTech, Nacka. Sweden) to measure responses to purified protein derivative (PPD) from *M.tb* (SSI, Copenhagen, Denmark), and pooled 15-mer peptides of ESAT-6 and CFP-10 (Peptide Protein Research LTD, Fareham, UK). The Project Licence enabling these studies was approved by the Ethical Review Process of Public Health England (PHE), Porton, Salisbury, UK and the Home Office, UK. Animals were housed in socially compatible groups and according to the Home Office (UK) Code of Practice for the Housing and Care of Animals Used in Scientific Procedures (1989), (now updated to Code of Practice for the housing and Care of Animals Bred, Supplied or Used for Scientific Purposes, December 2014), the NC3Rs, and the Guidelines on Primate Accommodation, Care and Use, August 2006 (NC3Rs, 2006). For procedures requiring removal from their housing, animals were sedated by intramuscular (IM) injection with ketamine hydrochloride (Ketaset, 100 mg/ml, Fort Dodge Animal Health Ltd, Southampton, UK; 10 mg/kg). None of the animals had been used previously for experimental procedures.

### 2.2 Clinical procedures

Animals were monitored daily for behavioural or clinical changes. Prior to blood sample collection, vaccination and euthanasia, animals were weighed, body temperature measured and were examined for gross abnormalities.

#### 2.2.1 BCG vaccination

Macaques were vaccinated intradermally in the upper left arm with 100 μl BCG Danish strain 1331 (SSI, Copenhagen, Denmark). BCG was prepared and administered according to manufacturer's instructions for preparation of vaccine for administration to human adults, by addition of 1 ml Sauntons diluent to a vial of vaccine, to give a suspension of BCG at an estimated concentration of 2–8 × 10^6^ CFU/ml.

#### 2.2.2 BCG infection

Macaques were infected intradermally in the upper right arm with 100 μl BCG Danish strain 1331 (SSI, Copenhagen, Denmark) at a concentration equivalent to an adult human vaccine dose (‘standard infection dose’) as described above, or at a concentration 5-fold greater (‘high infection dose’). For the high infection dose, 5 vials of BCG SSI vaccine were reconstituted with 0.2 ml of Sauntons diluent and combined to give a suspension of BCG at an estimated concentration of 1–4 × 10^7^ CFU/ml. In each case the exact location of the infection site was measured and recorded on a diagrammatic representation of the animal.

#### 2.2.3 Biopsy collection

Following sedation, the area of skin for biopsy was cleaned with 4% w/v chlorhexidine preparation (Hibiscrub, Regent Medical Overseas Ltd, Manchester, UK) and 1–2 ml of local anaesthetic (lignocaine, 10 mg/ml with adrenaline, 5 μg/ml, Xylocaine, AstraZeneca, Luton UK) injected subcutaneously in and around the BCG infection site. After 1–2 min, when the skin was fully anaesthetised, a 4 mm biopsy was collected using a disposable biopsy punch (William Needham & Associates, Duffield, UK), the piece of skin collected was transferred into a sterile cryovial and snap frozen. Following sample collection, pressure was applied to the skin with a gauze swab for 30 s–1 min and the site cleaned with a moist swab.

#### 2.2.4 Necropsy

Animals were anaesthetised, clinical parameters measured and skin biopsy collected from the site of BCG infection. The level of anaesthesia was deepened and blood samples were taken, prior to termination by intra-cardiac injection of a lethal dose of pentobarbitone sodium (Dolelethal, Vétoquinol UK Ltd, 140 mg/kg). A full necropsy was performed immediately, gross pathology assessed and the left and right axillary lymph nodes were collected and snap frozen.

### 2.3 Infection studies

A summary of the BCG infection studies performed is presented in Table 1.

**Table 1:** Summary of NHP BCG infection studies.

Study	NHP species	Number in study	BCG vaccinated (n)	BCG naïve (n)	Challenge dose	Time to sampling (days)	Samples taken
1	Rhesus	12	6	6	Standard	14	SB
2	Cynomolgus	32	0	8	Standard	14	SB + LN
			0	8	High	14	SB + LN
			0	8	Standard	28	SB + LN
			0	8	High	28	SB + LN
3	Cynomolgus	15	8	7	High	14	SB + LN
	Rhesus	12	6	6	High	14	SB + LN

^SB=skin biopsy, LN=lymph node^.

#### 2.3.1 Study 1: evaluation of initial human BCG infection model in NHPs

Twelve rhesus macaques were used to compare BCG-vaccinated and BCG-naïve animals with a study design successfully used in the early clinical trials of the BCG infection model in humans [16], [17]. Six animals received a BCG vaccination and 21 weeks later all 12 were infected intradermally with a standard dose of BCG. Twenty-one weeks is the standard interval used in *M.tb* challenge studies [28], [29] and therefore was chosen for this work. Fourteen days later, a biopsy of the infection site was taken and BCG quantified by culture on solid agar and quantitative polymerase chain reaction (qPCR).

#### 2.3.2 Study 2: development of the NHP BCG infection model

Both studies 2 and 3 were designed for the purpose of developing the BCG infection model further in NHPs. In study 2, thirty-two, BCG-naïve cynomolgus macaques were used for a comparison of BCG infection dose. Sixteen were infected with standard dose BCG and 16 were infected with high dose BCG. After 14 days, a skin biopsy of the infection site and draining axillary lymph nodes were harvested from eight animals from the standard dose group and eight animals from the high dose group. Biopsies and axillary lymph nodes were harvested 28 days post-infection from the remaining eight animals per group.

#### 2.3.3 Study 3: evaluation of the NHP BCG infection model

Using the optimal dose and time interval derived from Study 2, eight cynomolgus and six rhesus macaques received a BCG vaccination and 21 weeks later were infected intradermally with high dose BCG, along with seven BCG-naïve cynomolgus and six BCG-naïve rhesus macaques. Skin biopsies and axillary lymph nodes were taken 14 days post-infection.

### 2.4 Biopsy homogenisation and culture

Biopsies and axillary lymph nodes were processed as previously described [17], [19]. Briefly, samples were thawed and transferred to a dispomix tube (MACS) containing 1 ml sterile phosphate buffered saline (PBS). Tubes were loaded onto a dispomix machine (Thistle Scientific) and homogenised. The homogenate was then sonicated for 15 s. 100 μl neat homogenate and 100 μl of a 10^−1^ dilution in PBS, were plated in triplicate onto Middlebrook 7H11 agar (Appleton Woods Ltd.). Plates were incubated at 37 °C for 4 weeks before counting of colonies. The remaining biopsy or lymph node homogenate was stored at −80 °C for later DNA extraction.

### 2.5 DNA extraction

Biopsy or lymph node homogenate was thawed and BCG DNA from 200 μl homogenate was released using the tough micro-organism lysing kit (Precellys^®^) in a Precellys^®^24 machine at 6500 rpm for 3 × 30 s. Sample was transferred to a separate tube and 50 μl PBS used to wash remaining homogenate from the beads. Samples were then processed as previously described [19] and DNA was eluted in 400 μl AE buffer.

### 2.6 Quantitative polymerase chain reaction

Primers ET 1/3 (Forward - CCG CCG ACC GAC CTG ACG AC, Reverse - GGC GAT CTG GCG GTT TGG GG) modified by Minassian et al., were used for detection of BCG DNA. These are complementary to regions flanking the BCG deletion sequence, RD-1 and amplify a 196 bp fragment [30]. PCR reactions were carried out as previously described [19]. A standard curve was obtained by extracting BCG DNA from 1 in 10 serial dilutions of 5 pooled SSI vaccine vials in PBS and correcting for live BCG from the corresponding CFU counts on solid agar.

### 2.7 Statistical analysis

Statistical analyses were performed using GraphPad Prism. Data was not normally distributed, so one-way ANOVA (Kruskal-Wallis) and Mann Whitney U-tests were used to determine significant differences between groups. The Spearman's Rho test was used to determine correlations between amount of BCG recovered by culture and qPCR.

## 3 Results

### 3.1 Quantification of BCG from skin biopsies did not detect a difference between BCG-vaccinated and BCG-naïve rhesus macaques following infection with a standard dose of BCG

**Fig. 1:**
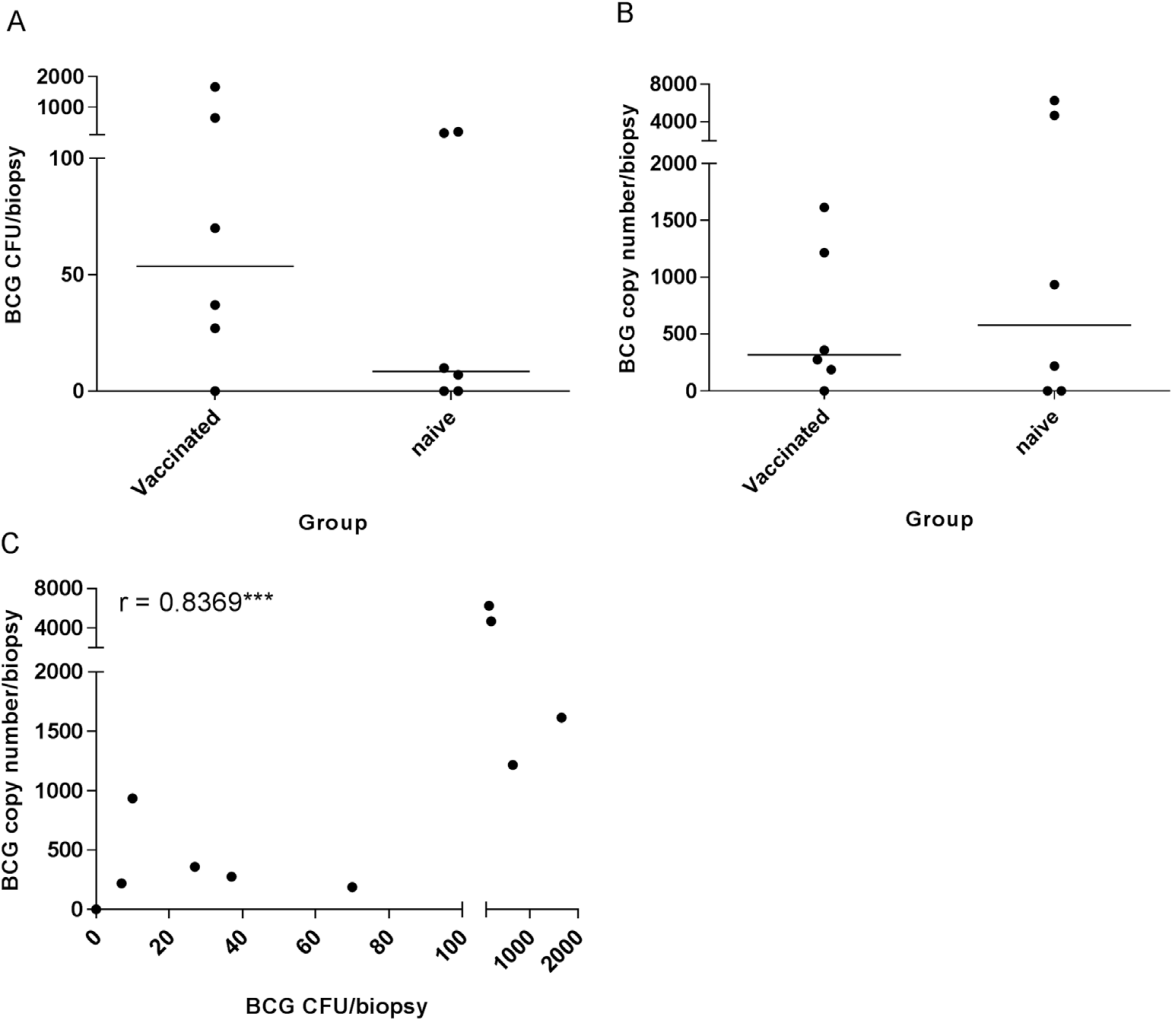
**Amount of BCG recovered from skin biopsies at the site of infection with standard dose BCG.** Amount of BCG detected by culture on solid agar (A) and qPCR (B) from BCG-vaccinated and BCG-naïve rhesus macaques (Study 1) and correlation between the two methods of detection (C). Dots represent individual animals, lines show median values and stars denote significance, *** = p < 0.001 (Spearman Rho).

BCG was detected from skin biopsies of the infection site in 5 out of 6 previously BCG-vaccinated and 4 out of 6 BCG-naïve rhesus macaques from study 1, by both culture on solid agar and qPCR (Fig. 1). Neither method of quantification detected a significant difference in the amount of BCG recovered between the two groups. The amount of BCG quantified from each biopsy was very low, with a median of 32 CFU/biopsy (range 0–1663), detected across the two groups by solid culture and 317 BCG copy number/biopsy (range 0–6268) by qPCR. BCG was undetected, by both culture and qPCR, in 25% (3/12) of NHP biopsies in this study, however, as only part of the sample was plated out and used for qPCR, there is a small possibility that these values might not be true 0s.

### 3.2 Infection with high dose BCG and 14 day sampling interval chosen for further studies

**Fig. 2:**
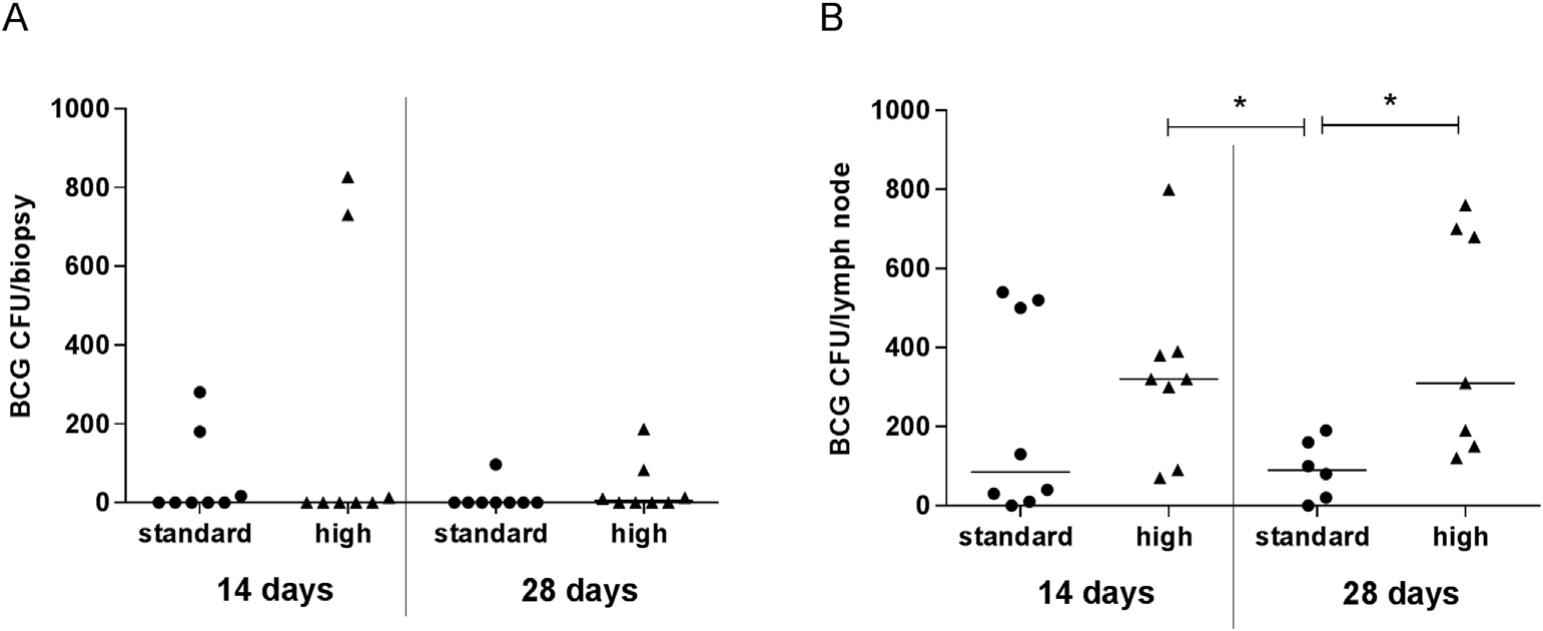
**Number of BCG CFU recovered after infection with standard or high dose BCG**. Number of BCG CFU recovered from skin biopsies (A) and draining axillary lymph nodes (B) of cynomolgus macaques infected with either standard or high dose BCG and sampled at either 14 or 28 days post-infection (Study 2). Dots represent individual animals, lines show median values and stars denote significance * = p < 0.05 (Mann Whitney).

Comparison of the numbers of BCG CFU recovered from skin biopsies of cynomolgus macaques, infected with standard and high dose BCG and sampled at 14 or 28 days post-infection (Study 2), showed no difference between the groups (Fig. 2A). Quantification of BCG from the draining axillary lymph nodes detected significantly higher numbers of BCG CFU in both of the high dose groups compared to the standard dose group who were sampled at 28 days post-infection (Fig. 2B) (Mann Whitney, p < 0.05). However lymph node data was not available for two animals in the standard dose group and one animal in the high dose group (all of which were sampled at day 28) due to contamination on the agar plates. Sampling at 14 or 28 days post-infection did not appear to have an effect on the amount of BCG recovered from the lymph nodes as statistical differences between the two standard dose groups or the two high dose groups were not seen (Mann Whitney, p > 0.05). Therefore, to be consistent with the human infection model, the 14 day sampling interval was chosen for study 3 with animals infected with high dose BCG.

### 3.3 Amount of BCG detected in draining axillary lymph nodes are lower in previously BCG-vaccinated cynomolgus macaques

**Fig. 3:**
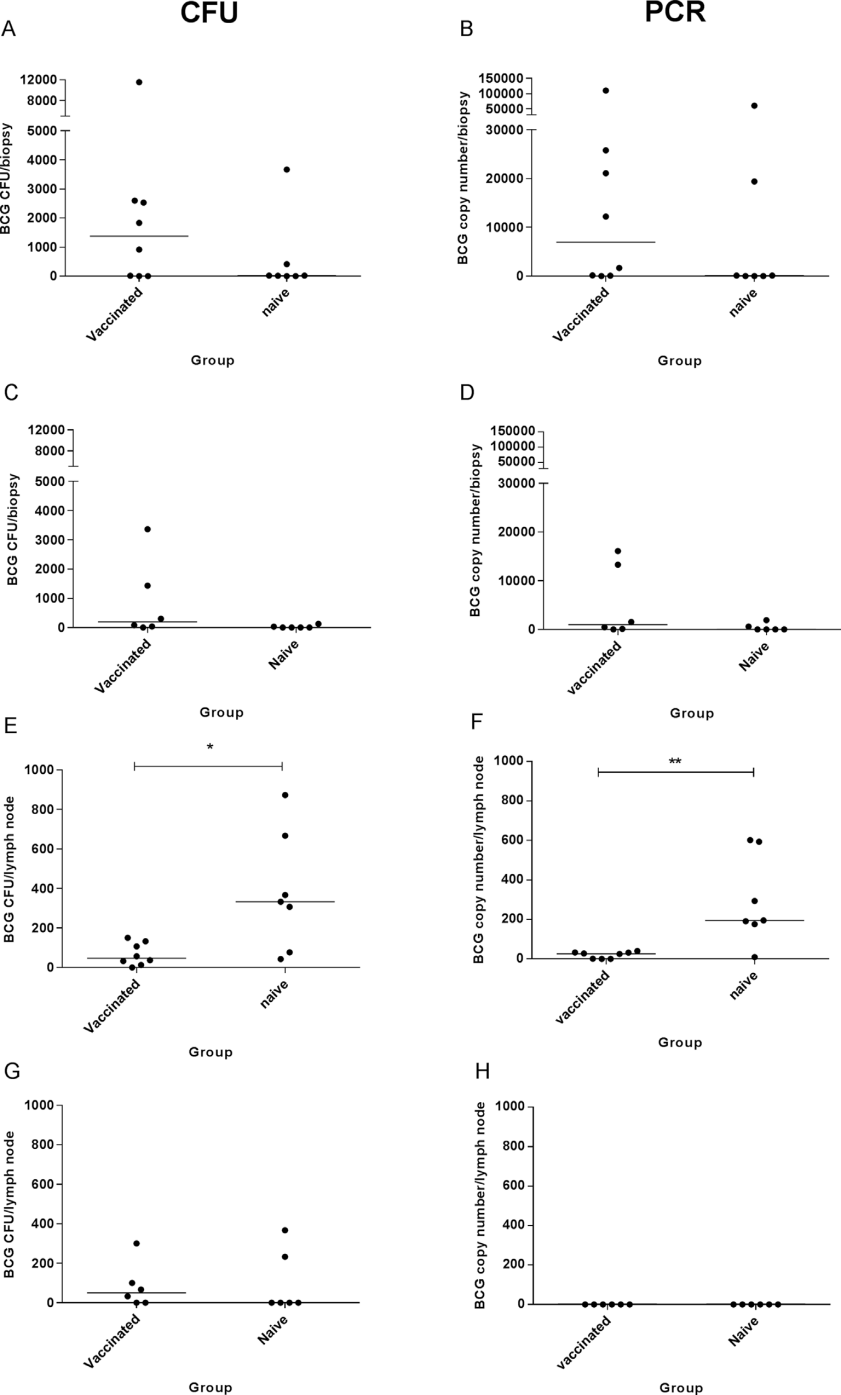
**Number of BCG CFU recovered in cynomolgus and rhesus macaques after infection with high dose BCG**. Number of BCG CFU or BCG copy number recovered from skin biopsies (A–D) and axillary lymph nodes (E–H) of cynomolgus (A, B, E and F) and rhesus macaques (C, D, G and H) 14 days post-infection with high dose BCG (Study 3). Dots represent individual animals, lines show median responses and stars denote significance, * = p < 0.05, ** = p < 0.01 (Mann Whitney).

Infection with high dose BCG did not result in a significant difference in the number of BCG CFU recovered from the skin biopsies of previously BCG-vaccinated and BCG-naïve animals in either the cynomolgus (Fig. 3A) or rhesus macaques (Fig. 3C). Quantification of BCG by qPCR also did not result in a significant difference between the groups (Fig. 3B and D). Quantification of BCG CFU from the axillary lymph nodes draining the site of infection, showed a significant reduction in the number of BCG CFU detected in the BCG-vaccinated group compared to the BCG-naïve group in the cynomolgus macaques (Fig. 3E) (Mann Whitney, p = 0.014). The same was true by quantification by qPCR (Fig. 3F) (Mann Whitney, p = 0.0059), In contrast to the cynomolgus macaques, there was no significant difference in numbers of BCG CFU detected in the draining axillary lymph nodes of BCG-vaccinated and BCG-naïve rhesus macaques (Fig. 3G). However, BCG was undetectable in 2 out of 6 lymph nodes in the BCG-vaccinated group and 4 out of 6 in the BCG-naïve group, compared to only 1 out of 15 in the cynomolgus macaques. BCG was undetectable by qPCR in all of the rhesus lymph nodes (Fig. 3H).

Assessment of gross pathology was completely normal for all animals in these studies.

## 4 Discussion

We have presented the results of a series of studies to develop and evaluate an NHP BCG infection model.

Quantification of BCG from skin biopsies of the infection site was not able to distinguish between BCG-vaccinated and BCG-naïve groups of animals after infection with standard or high dose BCG in either macaque species. This is in contrast to the human BCG infection model where, in two separate studies in healthy adults, significantly lower levels of BCG were detected in the skin biopsies of the BCG-vaccinated compared to BCG-naïve groups [16], [17]. The lack of a detectable vaccine effect from the skin biopsies of NHPs may be due to the lower amount of BCG recoverable from NHP compared to human skin biopsies and the higher percentage of biopsies in which BCG was undetectable. This discrepancy could be due to differences between humans and NHPs in the tissue structure of the biopsy, as macaque skin possesses less underlying fatty tissue than human skin, which may impact on BCG retention at the site, allowing dispersal from the infection site at a faster rate in NHPs than in humans.

In cynomolgus macaques, quantifying BCG from the axillary lymph nodes draining the site of infection can distinguish between the BCG vaccination status of the groups, with significantly lower levels of detection in previously BCG-vaccinated animals in a high dose infection model. The same was not true in rhesus macaques where BCG was detected in only 50% of the lymph nodes, suggesting that in this species, BCG does not disseminate so readily from the site of infection to the lymph nodes, highlighting the difference between the two NHP species in their response to BCG vaccination and supporting the finding that Mauritian cynomolgus macaques are less able to control *M.tb* than rhesus macaques [31]. This also emphasises the importance of characterising the response to BCG and *M.tb* in both species in more detail in order to select the most appropriate species to use to model human disease.

BCG vaccination has been shown to be partially protective against pulmonary *M.tb* infection in rhesus [29], [32] and cynomolgus [33], [34], [35] macaques. The results presented here from the high dose BCG infection model in cynomolgus macaques have shown a BCG vaccine effect similar to the partial protection seen with aerosol *M.tb* infection, as BCG growth was reduced in the BCG-vaccinated compared to the BCG-naïve group. These data provide important evidence to support the human infection model as it is not possible to validate against *M.tb* infection in humans. Demonstration of a BCG vaccine effect in rhesus macaques may be achievable after further development of the model. The ultimate validation of this model in NHPs would be to follow ID BCG infection with aerosol *M.tb* challenge and assess whether the results of the BCG infection are predictive of the outcome of the *M.tb* challenge. However, as the detectable vaccine effect was observed in the lymph nodes, this requires necropsy and therefore the same animals cannot go on to receive aerosol *M.tb*.

Models such this could also be used to help identify immune correlates of mycobacterial immunity, which could be targeted in further vaccine development. Peripheral blood mononuclear cells and serum samples from these studies have been cryopreserved and will be used to investigate cellular and humoral immune responses and perform mycobacterial growth inhibition assays (MGIAs) to assess correlations with the BCG infection outcome. This in turn will provide biological validation of the putative correlates such as MGIA, which may also have utility as an additional alternative to using virulent pre-clinical *M.tb* infection models to evaluate the efficacy of candidate TB vaccines.

Although an ID BCG infection model appears to be a valid surrogate for aerosol *M.tb* infection, there are some limitations. The natural route of *M.tb* infection is by aerosol. The aerosol delivery of BCG has been shown to be safe, immunogenic [36] and protective [37] in rhesus macaques and a clinical trial is currently ongoing in humans to assess aerosol BCG as a route of delivery, both as a potential route of vaccination and as a potential route of challenge in a controlled human infection model (clinicaltrials.gov ref. NCT02709278). Unlike *M.tb* and *M.bovis,* the genome of BCG does not possess the RD-1 region, which encodes the secretory proteins ESAT-6 and CFP-10, both important virulence factors [38]. Therefore, the efficacy of candidate vaccines which include these antigens may not be fully evaluated using this BCG infection model. However, an attenuated strain of *M.tb*, MTBVAC, is currently in phase 1 clinical trials [39] and such a strain could be considered for use in future controlled human infection models.

An infection model using BCG or an attenuated strain of *M.tb* would be a less severe way of testing new TB vaccines in NHPs and would comply with the NC3Rs criteria of improving the welfare of the animals as it would remove the requirement for animals to be exposed to virulent *M.tb* and to develop clinical signs of TB disease. They would also have a better quality of life as do not need to be confined to BSL3 facilities and therefore would benefit from a richer housing environment [25]. Furthermore, removing the expensive and restrictive BSL3 facilities needed for *M.tb* infection would increase the number of sites able to perform such studies and would be a more cost-effective, easier and potentially more high-throughput way of screening new TB vaccines. Both the NHP and human BCG infection models could provide a useful tool in TB vaccine development and aid in discovering and validating immunological correlates of protection.

## Funding

This work was supported by the Wellcome Trust and the Department of Health, UK. The views expressed in this publication are those of the authors and not necessarily those of the Department of Health. HMcS is a Wellcome Senior Clinical Research Fellow and a Jenner Institute Investigator.
